# Sperm DNA fragmentation index does not correlate with blastocyst aneuploidy or morphological grading

**DOI:** 10.1371/journal.pone.0179002

**Published:** 2017-06-07

**Authors:** Itai Gat, Katelynn Tang, Kevin Quach, Valeriy Kuznyetsov, Ran Antes, Melissa Filice, Khaled Zohni, Clifford Librach

**Affiliations:** 1 CReATe Fertility Centre, Toronto, Canada; 2 Sackler Faculty of Medicine, Tel Aviv University, Tel Aviv, Israel; 3 Pinchas Borenstein Talpiot Medical Leadership Program, Sheba Medical Center, Tel HaShomer, Ramat Gan, Israel; 4 Department of Obstetrics & Gynecology, University of Toronto, Toronto, Canada; 5 Department of Physiology, University of Toronto, Toronto, Canada; 6 Department of Gynecology, Women’s College Hospital, Toronto, Canada; The Babraham Institute, UNITED KINGDOM

## Abstract

High DNA fragmentation index (DFI) may be associated with poor outcome after IVF. Our aim was to determine whether DFI impacts blastocyst quality or clinical outcome. This retrospective study included 134 couples who underwent 177 IVF-ICSI and pre-implantation genetic screening (PGS) cycles during January 1^st^, 2014—March 31^st^, 2016 and had documented previous DFI. Group 1 (DFI>30%) encompassed 25 couples who underwent 36 cycles; Group 2 (DFI 15–30%) included 45 couples and 57 cycles; group 3 (DFI<15%) included 64 couples and 83 cycles. Male partners within group 1 were older (45.1 compared to 40.6 and 38.3 years, respectively, p<0.05), had higher BMI (32.4 compared to 26.6 and 25.8 respectively, p<0.05) and lower sperm count and motility (46*10^6^_/ml_ and 35.5%, respectively) compared to groups 2 (61.8*10^6^_/ml_ and 46.6%, respectively) and 3 (75.8*10^6^_/ml_ and 55.1%, respectively, p<0.05). Female parameters including ovarian reserve and response and embryo development were similar. Total numbers of biopsied blastocysts were 116, 175 and 259 in groups 1, 2 and 3, respectively. PGS for 24 chromosomes revealed comparable euploidy rate of 46–50.4%, with a similar morphological classification. No significant differences were found regarding pregnancy rates or pregnancy loss. It seems that DFI doesn't correlate with blastocyst aneuploidy or morphological grading.

## Introduction

Infertility is a common medical concern, which affects approximately one of every six couples attempting to conceive [1], and results from a male factor in up to 50% of cases [2]. Since the essence of sperm function is to deliver the paternal genome into the oocyte, chromatin integrity is essential to ensure not only fertilization, but also to support embryo development [3]. Sophisticated nuclear packaging mechanisms, such as replacement of histones with protamines, are performed throughout spermatogenesis [4] and result in highly condensed DNA in order to protect it and easily transport it within the female genital track. In their comprehensive review, Rathke et al described in detail the transformation process involving hyper acetylation of histones and incorporation of histone variants to loosen the nucleosomal structure followed by replacement of transition proteins by protamines, allowing tight DNA packaging into a higher-order structure. This sophisticated process is accompanied by the induction of DNA strand breaks [5]. Unfortunately, various processes may damage chromatin integrity leading to DNA fragmentation, such as apoptosis, enzymatically induced DNA breaks, radical oxidants species or gonado-toxic treatments [6].

Since the pioneering report of Evenson et al. (1980), who suggested that DNA integrity may be independent marker for male fertility [7], its possible impact on infertility has been investigated extensively. DNA fragmentation has been associated with decreased reproductive capacity in both natural and assisted conceptions [8]. A significant correlation was reported between DNA damage and paternal age, as well as abnormal semen analysis parameters [9,10]. Bungum et al. (2007) described significantly reduced pregnancy and delivery rates among infertile couples with a sperm DNA fragmentation index (DFI) > 30% compared to those with DFI < 30% in intra uterine insemination (IUI) cycles [2]. Despite the acceptable pregnancy rate achieved by IVF-ICSI for couples with high DFI, reduced pregnancy rates [11] [12] and increased risk for pregnancy loss [13] [1,14,15] remain major concerns. There is still controversy as to whether DFI should be investigated routinely or selectively among subgroups of infertile couples [16]. In addition, this test is not universally available in all fertility clinics.

Published data regarding the impact of sperm with high DFI on embryo development is limited. In their systemic review, Zini et al. (2011) stated that published studies are heterogeneous and that overall there is no consistent relationship between DFI and embryo morphology [17]. The only published study we could find that assessed preimplantation genetic screening (PGS) and sperm DNA fragmentation failed to demonstrate a correlation However, this study examined only day 3 embryos, used the more limited FISH technology and only involved 38 patients [18].

Embryo aneuploidy, which is the most common cause for early pregnancy loss [19], is mainly attributed to oocyte chromosomal missegregation [20]. However, that mechanism may not be relevant for pregnancy loss related to paternal high DFI, in which the DNA damage is mainly single or double strand breaks. Conversely, the higher probability of sperm aneuploidy and meiotic alternations in men with high DFI [6] could theoretically increase the risk for embryo aneuploidy. Therefore, our aim was to compare the rate of blastocyst aneuploidy (utilizing PGS for all 24 chromosomes) and morphological grading as well as clinical outcome among infertile patients with varying levels of sperm DFI.

## Material and methods

Approval for this retrospective study was obtained through the University of Toronto Research Ethics Board (#30444). Informed consent was not required due to study design, and patients’ privacy was kept continuously by reassuring anonymous data collection.

### Population

We evaluated the charts of all couples who underwent IVF-PGS between January 1^st^, 2014 and March 31^st^, 2016, and who had a documented DFI assessment prior to cycle initiation. All female partners underwent a standard, comprehensive fertility workup, including a detailed medical history, physical examination, laboratory evaluation, pelvic ultrasound, and a sonohysterogram to evaluate the uterine cavity and tubal patency prior to initiation of any treatment. Additional tests were performed as indicated. Basic male partner assessment included detailed medical history and semen analysis. DNA Fragmentation Index was performed at the judgment of the attending physician especially in cases of risk factors for abnormal DFI (ex. Smoking [21]) and abnormal semen analysis according to WHO criteria [22].

### Semen analysis and sperm DNA fragmentation assay

Semen samples were collected at CReATe Fertility Centre by masturbation. Semen analysis was performed on fresh sample according to the Examination and processing of human semen, fifth addition [22]. Samples for DFI assessment were frozen in two 500 μL aliquots until time of assessment. Aliquots were thawed at 37°C for three minutes and then diluted with TNE buffer (0.01 M TrisHCl, 0.15 M NaCl, and 1 mM of ethylenediaminetetraacetic acid (EDTA; pH 7.4) to 1–2×10^6^/mL. DNA fragmentation was evaluated by the sperm chromatin structure assay (SCSA) using flow cytometry with acridine orange as previously described [23]. Briefly, 200 μL of diluted semen was mixed with 400 μL of acid–detergent solution (0.1% Triton X-100, 0.15 M NaCl, and 0.08 N HCl (pH 1.2)) for 30 seconds, and then stained with acridine orange (AO; pH 6.0) for 3 minutes. Fluorescence measurements were collected on a duplicate of 8000 sperm per sample using a BD FACS Calibur (Becton–Dickinson, San Jose, CA, USA) from a 488 nm laser. DFI was expressed as a percentage of red fluorescence/ total (red + green) fluorescence using WinList 5.0. DFI was classified into three categories defined previously as low (15%), moderate (15%—30%) and high (≥30%), which corresponds to excellent, good to fair and poor fertility respectively [24].

### Ovarian stimulation

Controlled ovarian hyperstimulation for IVF included standard long agonist-based protocols, standard short antagonist-based protocols, and a minimal stimulation protocol for patients with low ovarian reserve, as previously described [25]. Exact dosing regimen was determined according to pre-stimulation assessment parameters such as age, AMH, antral follicle count (AFC), day 3 FSH, BMI, response to previous stimulation, infertility diagnosis (i.e. male factor, tubal etc.), and physician preference. Ovulation was triggered when lead follicles (>2) were ≥ 18 mm by ultrasound assessment with 5000–10,000 IU of chorionic gonadotropin (hCG) alone or with GnRH agonist (Lupron leuprolide acetate 0.4 cc). Patients with high ovarian reserve, who were at risk for ovarian hyper stimulation syndrome (OHSS), were triggered with GnRH agonist alone. Oocyte collection was performed 36 hrs post trigger. After ovum pick up, fertilization was achieved through ICSI of mature (metaphase II) oocytes, and *in vitro* embryo culture was performed as previously described [26,27].

### Blastocyst morphological evaluation and PGS

Morphological assessment was performed by experienced embryologists according to the Society for Assisted Reproductive Technology (SART) classification. Briefly, SART grade good was assigned for inner cell mass (ICM) grade A and trophectoderm (TE) grade A or B (AA or AB blastocysts); grade fair was assigned for ICM grade B and TE grade A, B or C (BB, BC, or BA blastocysts); grade poor was assigned for any ICM grade C (CC or CB blastocysts) [28].

Laser-assisted TE biopsy was performed at the blastocyst stage (day 5 or 6) as described previously [29] followed by cryopreservation. PGS analysis was performed for this study using array-comparative genomic hybridization (array-CGH) (BlueGnome Ltd, Illumina) in the CReATe PGS laboratory. Blastocysts without intact DNA for array-CGH analysis were excluded.

### Frozen embryo transfers (FET) and clinical outcome

Only frozen-thawed euploid blastocysts were transferred after endometrial preparation by exogenous estrogen (6–12 mg PO daily or 100mg every other day by dermal patches) followed by 6 days of progesterone (100mg IM QD or suppositories 200 mg 2-3/day) supplementation. The number of embryos transferred was determined by the number available as well as by patient age and clinical history such as previous ET failures, history of preterm delivery or abnormal uterus shape etc. Biochemical pregnancy was defined as positive serum β-hCG, but no gestational sac (GS) detected at 6 weeks. Clinical pregnancy was defined as the presence of an intrauterine GS. Ongoing pregnancy was defined as a viable fetus at 12 weeks gestation. Pregnancy loss was defined as spontaneous pregnancy demise before 10 weeks of gestation [30].

### Statistical analysis

The total population was divided into three separate cohorts according to the male’s documented DFI. Group 1 included couples with severely abnormal DFI (greater than 30%), group 2 was considered a moderately increased DFI level (15%-30%), and group 3 with a normal DFI (<15%) [13].

The primary endpoints were the proportion of euploid embryos (calculated as the number of embryos reported as euploid divided by the total number of embryos), as well as, blastocyst morphological classification. Additionally, all groups were compared based on the following characteristics: male parameters (age, BMI, lifestyle and semen analysis), female pre-stimulation evaluation (age, diagnosis, and ovarian reserve assessment), actual ovarian response (total dose of gonadotropins, E2 on trigger day, number of oocytes retrieved, number of mature MII oocytes), and embryonic development (fertilization rate, number of day 3 embryos and blastocysts available for biopsy).

Data was analyzed in the R statistical software (Version 3.2.2). For analysis, parametric continuous variables were expressed as mean values and standard deviation, and then compared with use of Analysis of Variance (ANOVA). Where required, non-parametric variables were analyzed with medians and interquartile range and compared using Fisher’s Exact test or Chi-Square Goodness of Fit test with post-hoc analysis. To investigate the relationship between DFI and the primary outcome, logistic regression analysis was used adjusting for the following covariates: male and female ages, DFI, motility, BMI, female AMH, AFC, FSH on day 3, total gonadotropins, number of retrieved eggs and number of blastocysts. All statistical tests were two-tailed and evaluated at the 0.05 level of significance.

## Results

**Fig 1:**
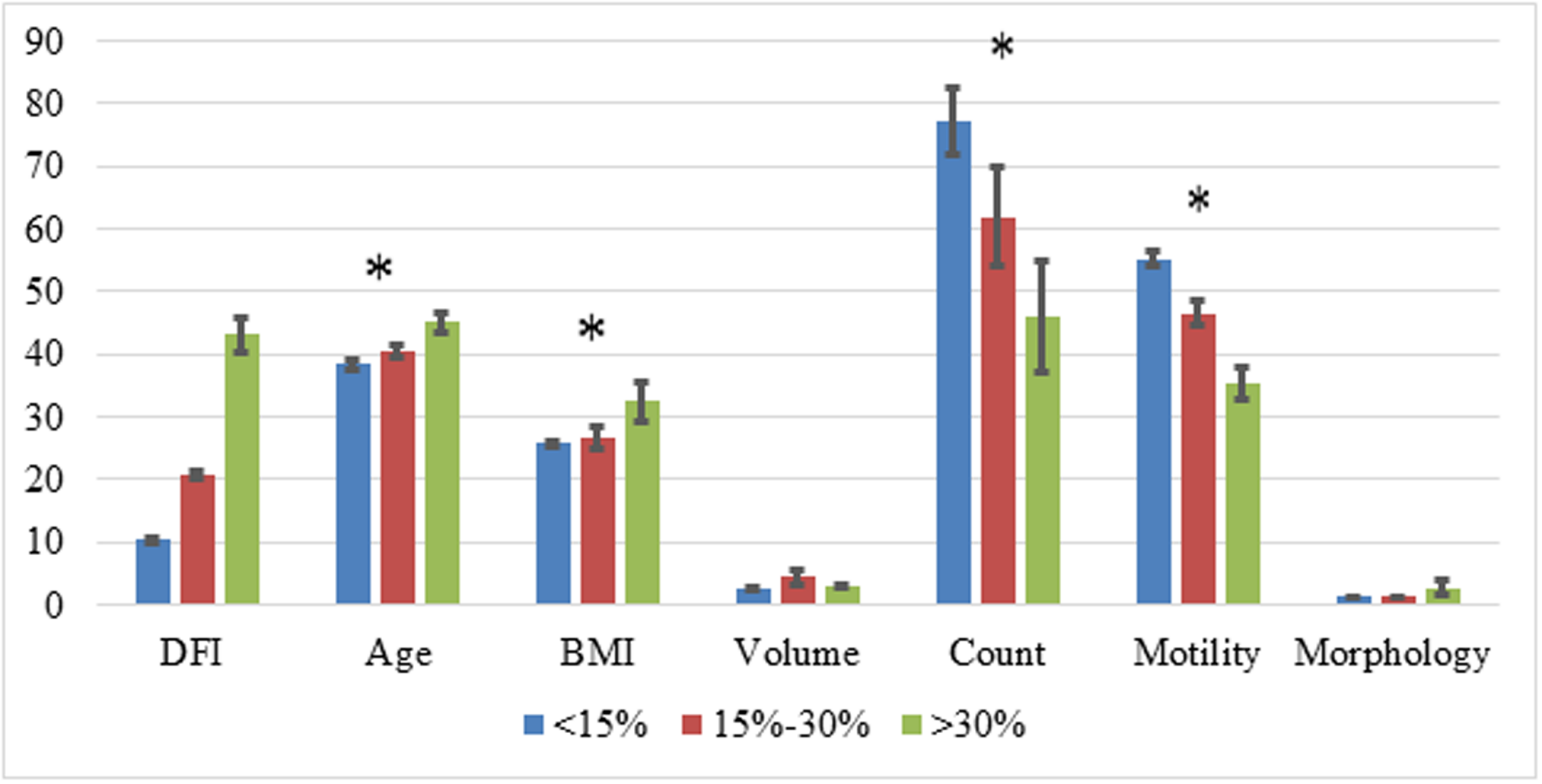
Male demographic parameters and semen analysis in multiple sperm DFI levels ^+^. ^**+**^ Each parameter is described by specific units in the Y axis as followed: DFI—%; age: years; BMI: Weight in Kilograms / (Height in Meters x Height in Meters); volume—ml; Count: millions sperm cells per ml; motility—% of motile spermatozoa; morphology—normal morphology according to Kruger criteria [22]. *p<0.05.

The current study included 134 couples who underwent 177 IVF-ICSI-PGS cycles. Group 1 (DFI>30%) comprised of 25 couples who underwent 36 cycles; Group 2 (DFI 15–30%) included 45 couples who underwent 57 cycles; and group 3 (control, DFI<15%), included 64 couples who underwent 83 cycles. This represented a mean of 1.3, 1.34 and 1.31 cycles per couple respectively (p>0.05). Male partners with high DFI were found to be older (45.1, range 32–62) in comparison to the other 2 groups (40.6 and 38.3 with ranges of 31–61 and 29–62 years respectively, p<0.05) and had a higher BMI (32.4 compared to 26.6 and 25.8, respectively, p<0.05). Additionally, patients in group 1 had significantly lower mean sperm count and motility (46*10^6^_/ml_ and 35.5%) compared to group 2 (61.8*10^6^_/ml_ and 46.6%, p<0.05) and group 3 (75.8*10^6^_/ml_ and 55.1%, p<0.05). Semen volume and sperm morphology did not differ between groups (Fig 1).

**Fig 2:**
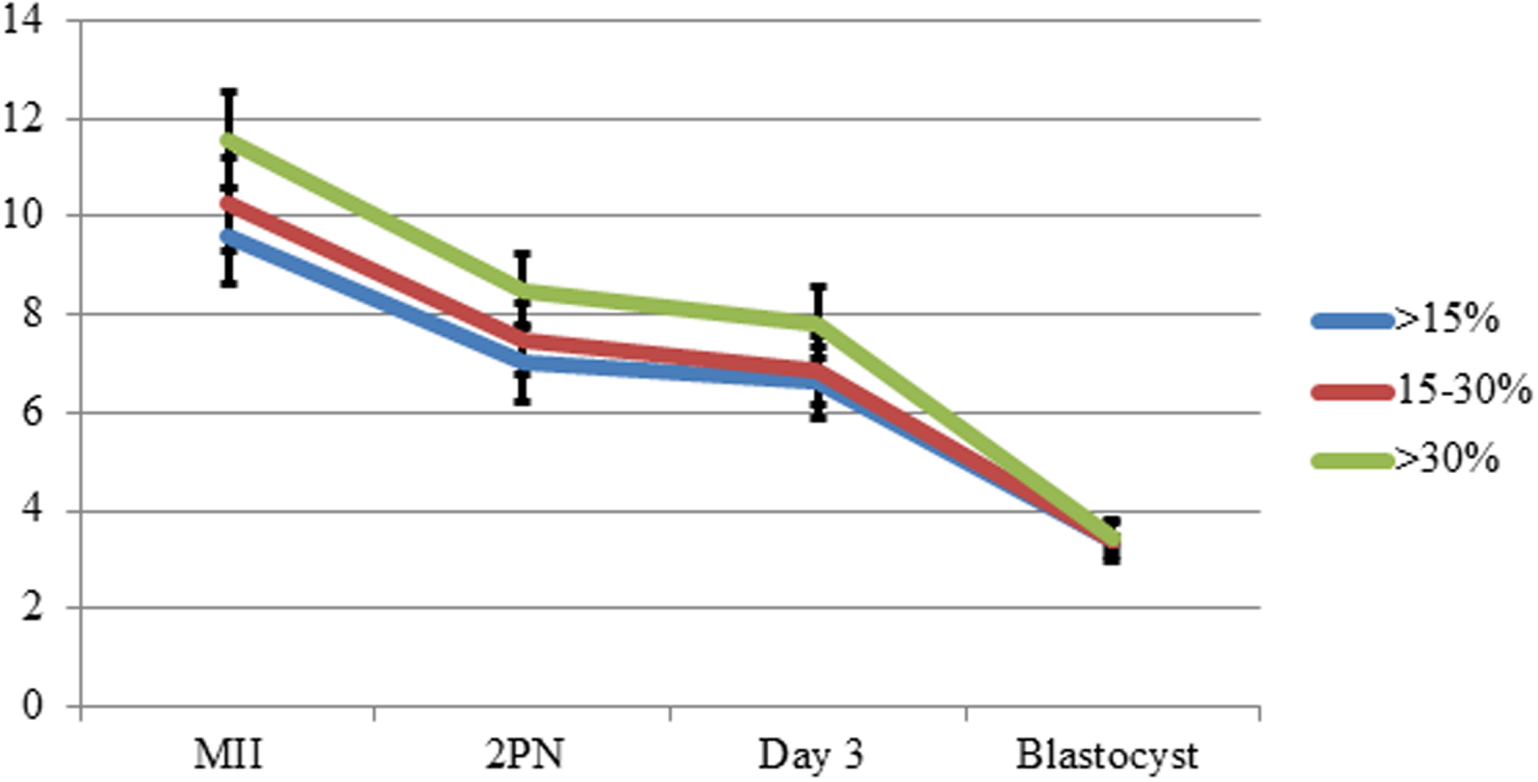
Pre-transfer embryo development (absolute numbers).

Female characteristics were similar between groups in both pre-stimulation assessment and actual ovarian response. No significant differences between groups were found for all ovarian reserve factors including age, AMH and AFC and ovarian response parameters (total gonadotropin dose, E2 on trigger day and number of retrieved eggs), although group 1 had significantly more IVF cycles compared to groups 2 and 3 (Table 1). Moreover, embryo development was similar between groups (Fig 2). Similar results were found in the two parameters used to assess paternal contribution to embryo development; fertilization rate (73±19%, 76.6±26% and 74.9±19%, respectively, p>0.05) and percentage of blastocysts compared to day 3 embryos (49.8±23%, 55±28% and 51±27% respectively, p>0.05).

**Table 1:** Comparable female characteristics between groups in both pre-stimulation parameters and ovarian response to hormonal stimulation.

		DFI>30%	15%<DFI<30%	DFI<15%	*p*
**N patients**		25	45	64	
**N cycles**		36	57	83	
**Median female age (range)**		37.3±4.2(27–45)	36.8±4.4(25–44)	37.5±4(28–45)	0.74
**BMI**		21.3±3.2	24.2±5.5	23±2.9	0.15
**AMH (median, pmol/L)**		30.2±30.1(19.8)	26.6±20.4(22.3)	28.9±30.6(17.9)	0.86
**AFC**		19.5±7.1	22±11.2	18.4±10.2	0.1
**IVF cycle number**		2.75±2.67	1.85±1.35	1.88±1.37	0.044
**Diagnosis (%)**	Male factor*	29.6	16.3	4.7	<0.01
	Decreased ovarian reserve**	63.0	34.9	18.8	0.19
	**Ovulation dysfunction**	48.1	27.9	28.1	0.23
	**Uterine and tubal**	14.8	14.0	23.4	0.08
	**Recurrent Pregnancy Loss**	3.7	16.3	7.8	0.61
	**Other**	7.4	4.7	7.8	0.21
**Total gonadotropins (units)**		4305±2162	4010±2345	3678±1917	0.34
**E2 on trigger day (pmol/L)**		13177±8878	12329±6825	10564±7606	0.21
**Retrieved eggs**		16.4±9.7	15.7±9.3	15.6±10.7	0.88
**MII**		11.6±5.6	10.4±6.7	9.8±6.9	0.35

* Abnormal semen analysis

** AMH<10pmol/L

The total number of blastocysts biopsied in group 1 was 116, 175 in group 2 and 259 in group 3. PGS using array CGH for all 24 chromosomes revealed a similar euploidy rate of 46–50.4% in all groups without significant difference (Table 2). Morphological classification of good (9–15%, p>0.05), fair (68–75%, p>0.05) and poor (16–18%, p>0.05) quality was also similar between the groups (Table 2).

**Table 2:** Blastocyst euploidy rate and morphological classification.

		DFI>30%	15%<DFI<30%	DFI<15%	*p*
**N blastocysts**		116	175	259	
**Euploidy rate (%)**		50.4	47.5	46.0	NS
**Morphological grading (%)**	**Good**	16	9	9	NS
	**Fair**	70	74	76	NS
	**Poor**	14	17	15	NS

Logistic regression analysis showed that both male and female ages were significantly associated with blastocyst euploidy rate (p = 0.001 and 0.006, respectively). DFI, other patients’ demographic parameters (such as BMI), semen analysis (motility), female AMH and AFC as well as stimulation doses and eggs retrieved did not correlate with blastocyst euploidy rate (Table 3).

**Table 3:** Logistic regression analysis of blastocyst euploidy.

	OR	Lower CI	Upper CI	*p* value
**DFI**	1.00	0.98	1.01	NS
***Male age***	**0.95**	**0.93**	**0.98**	**0.001**
**Motility**	1.00	0.99	1.02	NS
***Female age***	**0.91**	**0.85**	**0.97**	**0.006**
**Female BMI**	1.01	0.96	1.07	NS
**AMH**	1.00	0.99	1.01	NS
**AFC**	1.01	0.99	1.02	NS
**FSH on E2<200**	1.03	0.95	1.10	NS
**Total gonadotropins**	1.00	1.00	1.00	NS
**# retrieved eggs**	1.01	0.99	1.02	NS
**Blastocysts**	1.03	0.98	1.08	NS

Eighty eight cycles (49.7%) resulted with embryo transfer: 21 (60%) in group 1, 30 (51.7%) in group 2 and 37 (44%) in group 3. Although the average number of embryos transferred was higher among the high DFI group (1.3) compared to intermediate and normal DFI (1.2 and 1.1, respectively), that difference was not statistical significant. Biochemical, clinical and ongoing pregnancy rates were similar between the groups. Pregnancy loss was found as 8%, 24% and 12% in the three groups, respectively, without significant difference (Table 4).

**Table 4:** Clinical outcome.

	DFI>30%	15%<DFI<30%	DFI<15%	*p*
N ET*	21	31	36	
**Embryos per transfer (mean±SD)**	1.3±0.5	1.3±0.4	1.1±0.3	NS
**Clinical pregnancy (%)**	12 (57)	17 (55)	17 (46)	NS
**Ongoing pregnancy (%)**	11 (52)	13 (42)	15 (41)	NS
**Pregnancy loss (%)**	2 (16)	4 (24)	2 (12)	NS

* Embryo transfers. Median and range of transferred embryo were 1 and 1–2, respectively, in all groups.

## Discussion

Embryo development and implantation are dependent, at least partially, on the integrity of sperm DNA [17]. High levels of sperm with fragmented DNA is associated with diminished fertilization and embryo development [13], unless integrity damage exceeds certain threshold [31]. Although multiple studies have reported negative effect of high DFI on reproductive outcome in IVF-ICSI [13,15], the exact mechanism(s) by which DFI impairs fertility is not clear. In the current study we focused on the impact of sperm DNA damage on pre-implantation embryonic ploidy and morphological grading. Additionally, post-implantation embryonic development was evaluated by pregnancy rates and pregnancy loss.

Our sample size included 134 couples who had 177 IVF-ICSI cycles in which PGS was performed in 405 blastocysts. DFI was not associated with impaired embryo quality in both genetic and morphological evaluations. Moreover, DFI had no impact on clinical outcome after euploid blastocyst transfers. In the same line, Cissen et al have recently reported in their meta-analysis that DFI has limited capacity to predict the chance of pregnancy in the context of medical assisted reproduction [32]. However, the current study includes not only outcome measurements but also comprehensive chromosomal screening (CCS) by a-CGH. Theoretically one would argue that the possible negative impact of abnormal DFI on pregnancy rate has been biased and removed by PGS—aCGH performance, which resulted with only euploid blastocysts transfers. However, since no correlation between DFI and euploidy rate was demonstrated we believe this arguement is not relevant.

Similar DFI-related findings were published by Bronet et al. (2012), who reported comparable embryo euploidy rates in different DFI levels [18]. However, their study, had several limitations. First, it included only 38 couples (30 with RPL and 8 RIF) with only one sample in the cohort had a DFI>30%. Our study, on the other hand, has a much larger sample size and includes many samples with high DFI levels. Secondly, embryo biopsy was performed on day 3, which is considered to be higher risk and less accurate compared to the blastocyst TE biopsies [33] performed in this study. Thirdly, the authors performed FISH for only a subset of 9 chromosomes, while our report included a-CGH for all 24 chromosomes. In spite of the advantages of the current methodology, certain limitations are still important to be mentioned. Most importantly, the examined trophoectoderm is not a homogeneous representation of the inner cell mass (ICM) [34]. Mosaicism remains a major challenge to interpret PGS results since TE mosaicism may be present in at least half of all embryos [35]. The clinical importance of mosaicism has been emphasized by Greco et al, who transferred mosaic embryos after PGS performance, resulting with delivery of healthy babies in one third of cases [36]. However, we feel that the conclusion of our manuscript is still relevant since possible paternal-related aneuploidy will result with complete aneuploidy rather than mosaicism. Moreover, PGS was performed to estimate the occurrence of aneuploidy in a population of embryos.

The literature focused on a possible correlation between DFI and embryo morphology grading is heterogeneous due to varying stages of development (day 2, 3 or 5) and differences in grading criteria [17], resulting with conflicting conclusions [37,38]. Similar to PGS findings, blastocyst grading to good, fair and poor quality was similar between the groups. While previous reports demonstrated higher risk for arrested [39] or slower development to blastocysts in couples with high DFI [40], to the best of our knowledge this is the first assessment of DFI impact on blastocyst grading.

One of the crucial milestones during embryo development is the activation of embryonic genome between 4 and 8 cells stage [41]. This likely explains why semen having a high percentage of sperm with fragmented DNA appears to be associated with a reduced rate of blastocyst formation from day 3 of development [42]. Our data did not support this notion as there was no significant difference in blastocyst formation between the three groups (Fig 2). This could possibly be explained by oocyte DNA repair mechanisms [43]. Egg capacity to repair sperm DNA damage is strongly related to female age [1]. Since there was no difference in female age among the groups, the oocytes in each group would be expected to have an equal capacity to repair sperm DNA fragmentation, which may explain the equal euploidy rate.

Post-implantation embryo development was assessed by clinical outcome. Compared to previous reports of impaired outcome and especially higher pregnancy loss after IVF and ICSI cycles due to high DFI [44,45], pregnancy rate and pregnancy loss in the current study were not significantly different between the groups. We found comparable pregnancy rates such as ongoing pregnancy rate of 41–52% without significantly higher pregnancy loss related to high DFI. To the best of our knowledge, this is the first research which includes only euploid blastocysts transferred after PGS for 24 chromosomes. The lack of correlation between DFI and pregnancy loss may be related to small sample size of ET cycles. However, in the same line with the current frozen ET results, previous reports didn’t demonstrate correlation between sperm DFI and clinical pregnancy, biochemical pregnancy and miscarriage rates after frozen ET of both day 3 and day 5 embryos [46]. Therefore the lack of consensus regarding the impact DFI on IVF-ICSI outcome is far from being clear.

Although paternal age was not the focus of our study, its significant correlation with embryo euploidy (OR 0.95, CI 0.93–0.98, *p* = 0.001) was unexpected. This relationship held up even when controlling for maternal age. The detrimental effect of advanced maternal age on reproductive outcome by increased embryo aneuplidy is well known [47,48] and demonstrated in the current study. In contrast, the effect of paternal age has been comparatively less studied and there is no common consensus on its role in reproductive success [49]. While the relationship between advanced paternal age and specific genetic diseases (achondroplasia, Apert syndrome, myosis ossificans and Marfan syndrome) has been reported [50], its possible correlation with sperm numerical abnormalities is controversial. Although advanced paternal age is associated with impaired semen analysis and high DFI [51,52], and poor sperm parameters is associated with sperm aneuploidy [53], an association between paternal age and aneuploidy is still uncertain [54]. Importantly, our findings should be addressed with caution since we could not exclude the possibility that paternal age was associated with aneuploidy due to their female partners, since older men tend to marry older women. The impact of paternal age not only on semen parameters but also on embryonal development and euploidy remains puzzling in the era of late parenthood.

Strengths of this study include the size of the cohort and the use of comprehensive chromosome screening on blastocyst stage embryos. However, there are several limitions of the study. Firstly, although these findings are valuable, they are limited by selection bias, as we included only infertile couples in whom DFI was assessed. That cohort may not accurately reflect the general population who undergo IVF. We plan to examine patients undergoing cycles with young egg donors who underwent PGS testing in future studies, to further isolate the impact of DFI alone on embryo aneuploidy. Secondly, we also acknowledge selection bias regarding female patients due to PGS performance as an inclusion criteria. Since we see PGS primarily as an embryo selection tool, its usage is usually only recommended in cycles which produce a relatively large cohort of blastocysts. Therefore, patients undergoing PGS usually have a higher ovarian reserve than patients who do not [55]. As a consequence, it is not surprising that our population was characterized with a higher than average mean AMH (mean 26.6–30.2 pmol/L) a high mean number of retrieved eggs (15.3–16.4) and developing embryos up to blastocyst stage (average of 3.4 in all groups). However, that bias does not likely interfere or limit the study’s conclusion regarding the lack of impact of sperm DFI on blastocyst euploidy and morphological grading. Thirdly, our morphological grading did not include time lapse morphokinetic evaluation, which may reveal differences between the groups [40]. Fourthly, the relatively small sample size of cycles with ET prevents definitive conclusions regarding the possible impact of DFI on clinical outcome in cases of euploid embryo transfers. Lastly, although criteria to perform DFI in our clinic is quite uniform among physicians, patient selection may be seen as a confounder of the study. Additional cofounder may arise from semen samples processing. While SA was examined on fresh samples only, SCSA has been performed mainly for frozen—thawed samples. Boe-Hansen reported that incubation of the semen samples on ice postthaw has significant impact on DFI [56]. Although such an impact would damage all samples, we can't exclude the possibility that samples with abnormal DFI may be more sensitive to freezing-related damage.

In conclusion, we found no correlation between the level of sperm DNA fragmentation and blastocyst quality or pregnancy outcomes.

## Supporting information

S1 File Data set. (XLSX)

S1 Table Summarizing table for the median and range of key parameters. (DOCX)
