# Supplementary material for: Sperm DNA fragmentation index does not correlate with blastocyst aneuploidy or morphological grading
Source: PLoS One. 2017 Jun 7;12(6):e0179002. doi: 10.1371/journal.pone.0179002 (PMC5462460; doi:10.1371/journal.pone.0179002)
Supplement: S1 Table — (DOCX) [file pone.0179002.s002.docx]

***Supporting information***

**S1 Table 1.** Summarizing table for the median and range of key parameters

|  | **>30%** | | **15%-30%** | | **<15%** | |
| --- | --- | --- | --- | --- | --- | --- |
|  | **Median** | **Range** | **Median** | **Range** | **Median** | **Range** |
| **Male age** | 46 | 32-62 | 40 | 31-61 | 37 | 29-62 |
| **Female age** | 37 | 27-45 | 38 | 25-44 | 37 | 28-45 |
| **AMH** | 19.8 | 2.5-132.5 | 22.3 | 2.4-81.7 | 17.9 | 2.8-150 |
| **AFC** | 20 | 14885 | 22 | 5-52 | 15.5 | 4-50 |
| **Total gonadotropins dose** | 3750 | 1575-10875 | 3562.5 | 1200-12375 | 3100 | 1425-8025 |
| **E2 on trigger day** | 10183 | 3763-36293 | 10911 | 1504-27379 | 8586.5 | 1122-38404 |
| **Number retrieved eggs** | 13 | 4-30 | 14 | 1-47 | 13 | 3-67 |
| **Number of MII** | 11 | 4-26 | 9 | 1-39 | 8 | 2-43 |
| **2PN** | 8 | 1-20 | 7 | 1-29 | 6 | 2-35 |
| **Fertilization rate (%)** | 72.7 | 33-87 | 74.7 | 29-100 | 77.5 | 0-100 |
| **Blastocyst #** | 3 | 01-8 | 3 | 0-9 | 3 | 0-18 |
| **Blastocyst #/day3 (%)** | 50 | 10-100 | 51.9 | 10-100 | 50 | 0-100 |
| **# of transferred embryo** | 1 | 1-2 | 1 | 1-2 | 1 | 1-2 |
| **Clinical Pregnancy** | 1 | 0-1 | 1 | 0-1 | 0 | 0-1 |
| **Ongoing pregnancy** | 1 | 0-1 | 0 | 0-1 | 0 | 0-1 |
